# Taking the history in patients with swallowing disorders: an international multidisciplinary survey

**DOI:** 10.1007/s00261-016-0931-4

**Published:** 2016-10-11

**Authors:** Martina Scharitzer, Peter Pokieser, Michaela Wagner-Menghin, Ferdinand Otto, Olle Ekberg

**Affiliations:** 1 Department of Biomedical Imaging and Image-guided Therapy, Medical University of Vienna, Waehringer Guertel 18-20, 1090 Vienna, Austria; 2 Unified Patient Project, Teaching Center, Medical University of Vienna, Vienna, Austria; 3 Teaching Center, Medical University of Vienna, Vienna, Austria; 4 Department of Neurology, University Hospital Salzburg, Salzburg, Austria; 5 Department of Translational Medicine, Division of Medical Radiology, Skåne University Hospital, Malmö, Sweden

**Keywords:** Deglutition, Deglutition disorders, Medical history-taking, Questionnaire

## Abstract

**Purpose:**

Clinical assessment of swallowing disorders (dysphagia) requires accurate and comprehensive medical history-taking to further tailor the diagnostic work-up, but functional health care questionnaires show a large variability and various limitations. The aim of this study was to assess the way in which international swallowing experts from various disciplines asses swallowing problems in order to improve the radiologist´s ability to take a thorough medical history in this specific patient group.

**Methods:**

A two-step Delphi method was used to collect swallowing experts’ ways of taking the medical history in patients with swallowing disorders. The questions obtained in a first interview round were pooled and structured by dividing them into general and specific questions, including several subcategories, and these were scored by the experts in a second step based on to their clinical relevance.

**Results:**

Eighteen experts provided 25 different questions categorized as general questions and 34 dimension-specific questions (eight attributed to ‘suspicion of aspiration,’ 13 to ‘dysphagia,’ six to ‘globus sensation,’ four to ‘non-cardiac chest pain,’ and three to ‘effect of life.’) In the second interview round, the experts´ average predictive values attributed to those questions showed the varying importance of the presented items. Seven general and 13 specific questions (six of them attributed to ‘effect on life’ and seven ‘others’) were also added.

**Conclusions:**

This collection of questions reflects the fact that a multidisciplinary approach when obtaining the medical history in patients with swallowing disorders may contribute to an improved technique for performing a symptom-oriented medical history-taking for radiologists of all training levels.

Swallowing disorders (dysphagia) are an increasing cause of disability and morbidity [1, 2]. They can be found in 12% of hospital admissions in an acute care setting and in up to 60% of nursing home populations [3]. These disorders may have severe consequences on the patients’ health status by affecting their ability to eat and drink [4]; thus, identifying the cause of these disorders quickly is vital in expediting treatment and rehabilitation. Although dysphagia is simply defined as the medical term for difficulty in swallowing, its variations in pathophysiology mandate the ability to comprehensively explore the symptoms.

Varying diagnostic examinations, including radiologic and non-radiologic techniques, are used to evaluate patients with swallowing disorders. Oropharyngeal and esophageal dysphagia are best evaluated with videofluoroscopic and fiberendoscopic imaging [5–7]. However, procedure planning, follow-up imaging, and diagnosis often rely on integration of pertinent patient history. In addition, the integration of clinical information is mandatory for a comprehensive interpretation of the radiological findings.

The relevance of history-taking has long been established. About 60% to 80% of diagnoses in internal medicine are derived conclusively from an initial comprehensive interview [8, 9]. Murray et al. showed that a detailed exploration of dysphagia symptoms, together with learning about a patient´s demographic background, helps in differentiating between malignant vs. peptic strictures [10]. Performing history-taking well means to master the communication aspect simultaneously with the clinical reasoning aspects of the situation [11]. Suboptimal communication has been shown to result in therapeutically suboptimal decisions. For example, in a study on the treatment of early prostate cancer, about one-third of the patients received treatment that did not consider their preexisting dysfunctions [12]. Less than optimal history-taking may also lead to less compliant patients, as evidenced by a report that revealed that poorly communicating doctors have a 1.47 times greater risk of non-adherence compared to patients who have a doctor who communicates well [13]. Suboptimal clinical reasoning during medical history-taking may also result in an incorrect attribution of symptoms or incorrect further diagnostic work-up. Melleney et al. reported that 15% of patients referred to a specialized gastroenterology unit for the evaluation of dysphagia had no swallowing problems [14].

To competently obtain the clinical history in a problem-solving manner, a thorough knowledge of the causes of each symptom and the symptoms of each disease is mandatory [15]. Clinically experienced physicians more often use this style of history-taking. Several authors have stated that medical students typically prefer diagnostic tests and methods for making a diagnosis, whereas experts in the field rely more on the medical history [16]. Health care professionals of various disciplines provide important input from their individual perspectives for the multidisciplinary management of patients with often complex issues. McCullough et al. [17] evaluated the interjudge reliability for questions that were typically employed during medical history-taking and called for more precise definitions of clinical examinations in patients with swallowing disorders. There are a small number of published functional health care questionnaires with various limitations that reinforce the importance of further research in this area [18]. The role of radiologists in taking the history varies greatly, although knowledge of the overall clinical situation is mandatory for an optimal planning, implementation, and interpretation of the radiological study.

The aim of this study was to enhance the radiologist´s ability to take a medical history-taking in patients with swallowing disorders by providing the core of experts´ knowledge and experience in this regard. Therefore, a group of interdisciplinary international swallowing experts participated in a two-step, questionnaire-based process.

## Materials and methods

A two-step Delphi method [19] was used to determine swallowing experts’ ways of taking the medical history in patients with swallowing disorders. In such a structured communication process, an expert panel first provides opinions about a key question. These opinions are then pooled and structured by the researchers and the result is fed back to the expert panel. In a second step, the expert panel rates each statement’s importance for answering the key question.

### Swallowing experts

For step 1, a multidisciplinary expert panel was identified and selected based on their clinical background and their experience in patients with swallowing disorders, as evidenced by participation on the board or being invited to speak at the first congress of the European Society of Swallowing Disorders (ESSD) in Leiden/the Netherlands. In the first step, 18 swallowing experts were approached and consented to participate in an interview about their way of taking the medical history. This expert panel consisted of 14 out of 17 members of the original board of the ESSD and 4 invited speakers, attributable to the following medical speciality: eight swallowing experts from otolaryngology departments, four from radiology departments, four members of departments of speech and language pathology, one from an internal medicine department, and one from a department of surgery. The participants came from the following countries: Australia, Austria, Belgium, Canada, France, Germany, Greece, Ireland, Italy, the Netherlands, Spain, Sweden, the UK, and the USA. The experts reported that they saw about 500 patients per year, on average, ranging from 100 to 1500.

For the second step, 25 experts with a comparable clinical backgrounds and experience in patients with swallowing disorders and a comparable number of patients investigated per year were contacted and invited to participate in a ranking of identified history-taking topics for swallowing disorders (e-mail: *x* = 9; contacted personally: *x* = 16 during the ESSD congress in 2014). Eleven of these experts (44%) had also taken part in step 1. There were 8/25 experts in the field of ENT, surgery (*n* = 6), radiology (*n* = 5), internal medicine (*n* = 3), speech and language pathology (*n* = 2), and neurology (*n* = 1).

### Materials

For step 1, an interview manual with 14 items was designed to serve as a guiding tool for the open-ended interviews (Appendix 1).

For step 2, the themes and their respective questions covered during history-taking in patients with swallowing problems, as identified in step 1, were listed. Questions were accompanied by a scale of 1–6, where 1 represented a very important question for every patient with swallowing disorders, used in 100% of interviews, and 6 represented a rare question used only in specific situations in <10% of interviews. At the end of each theme, ‘missing questions attributable to the preceding theme’ could be entered as text. In addition, the experts were asked to indicate whether they approved of the given structure of the questions and whether they could imagine using a questionnaire based on this structure for their future work.

### Procedure

For the first step, swallowing experts were interviewed by an experienced radiologist (P.P.) and a medical student in his final year (F.O.). During the first round, 18 interviews lasted for about 25–30 min each. Notes, taken immediately during the interview, were transcribed and subsequently mailed to each swallowing expert for review and specification.

Swallowing experts were emailed the results of step 1 and invited to participate in a questionnaire where they rated the importance of each question.

### Analysis

Step 1: In order to pool and structure the answers to the question “How do you take the history in patients with swallowing disorders?,” we performed a directed content analysis [20] (M.S., P.P., M.W-M.) with deductive category application [21]. The first coding dimension, ‘general questions’ and its four subcategories, was derived by drawing on common models on which history-taking is based [22]. Thus, general questions included questions classified as ‘opening the encounter,’ ‘symptom analysis,’ ‘nutrition,’ and ‘consistency.’

The second coding dimension, “specific questions for differential diagnosis,” was derived based on dysphagia symptoms, as well as on the most common symptoms associated with swallowing disorders, such as suspicion of aspiration, globus sensation, and non-cardiac chest pain [23], as well as specific questions attributable to the effect on the patient’s quality of life, and, finally, other questions not allocated to any of the above groups.

Frequencies within each coding dimension and each subcategory were counted. The percentage of the expert sample that provided items for each subcategory was also determined.

Step 2: To determine the importance of each question given in step 1, the scores of each rating of the questionnaire were averaged over the total number of experts to evaluate the average predictive value. The results were entered in a spreadsheet application and were expressed as percentages of the responses.

## Results

Step 1—Answers to key questions; directed content analysis.

The 18 experts provided 25 different items, categorized as general questions. Two items were assigned to ‘open questions,’ five to ‘symptom analysis, ’twelve to ‘nutrition,’ and six to ‘consistency.’ The dimension-specific questions about swallowing disorders comprised 34 different items: most of them attributable to ‘dysphagia’ (*n* = 13), eight to ‘suspicion of aspiration,’ six to ‘globus sensation,’ four to ‘non-cardiac chest pain,’ and three to ‘effect on life.’

Step-2—Rating the importance of themes and questions during medical history-taking in patients with swallowing disorders.

Table 1 shows all general questions and the average expert estimated values attributed to those questions by the specialists interviewed with regard to obtaining a clinical history in patients with swallowing disorders. Table 2 shows the specific questions including the average expert estimated values.

**Table 1:** General questions

Theme: Opening the encounter with open question/patient’ narrative	
What is the main problem?	1.44
Why are you here?	1.92
Theme: Symptom analysis	
When did the problem begin?	1.2
How did the problem start?	1.76
Have your symptoms always been like this?	2.5
Are your symptoms intermittent or consistent?	1.4
Has the symptom been constant for the last year/month/week?	1.96
Is there a trigger for the symptoms?	*
Did the problem start with solid food or with liquids?	*
Do your symptoms occur at the beginning or later during a meal?	*
Is swallowing painful?	*
Theme: Nutrition	
Does the problem affect solids?	1.17
Does the problem affect liquids?	1.17
Do you avoid certain kinds of food?	1.46
Which kind of food is the best/worst?	1.96
Are you able to swallow solid food/Bread crust/meat?	2.2
Do you have to cut your food into small pieces?	2.52
Do you need to take a drink after swallowing solid food?	2
Did you spare any kind of food before the problem occurred?	3.5
Do you need more time for your meal than others?	2.08
In which position do you eat?	3.44
In which position do you sleep?	4.44
Are you able to swallow spontaneously?	3.33
Subtheme: Consistency	
Is the problem depending on the consistency of the bolus?	1.92
What consistency of food is most difficult for you to swallow?	1.76
Is there any food consistency, which may be eaten/swallowed without impairment?	2.12
Do you avoid certain kinds of food consistencies?	2.12
Do carbonated liquids or sorbets ease your symptoms?	3.32
Do you have to eat modified food?	1.96
Is the temperature of any influence?	*
Do you have difficulties to swallow dispersible food (rice) or double consistencies food?	*
Drinking when food gets stuck relieves symptoms/has no effect/increases discomfort?	*

Numbers indicate the average expert estimated value assigned by the swallowing experts of the second assessment round

* Indicates questions, that were added as missing in the second interview step, but not rated by all interviewed

**Table 2:** Specific questions

Basic pathway I: Dysphagia	
Does food get stuck in your throat while you eat?	1.33
Where do you feel food sticking throat/thorax/stomach?	2
Does food come back into your throat/mouth after you swallowed?	1.43
Do you have to cut your food into small pieces?	2.65
Do you need to take a drink after swallowing solids?	2.09
Do you have to vomit occasionally? If so, when?	3.22
Do you suffer from too much saliva?	3.13
Do you have problems swallowing your saliva?	1.96
Do you suffer from hoarseness?	2.13
Do you suffer from a gargling voice?	2.5
Is there saliva on your pillow when you wake up in the morning?	4.04
Do you have hearing impairments?	4.38
Do you suffer from any neurological impairment?	2.21
Basic pathway II: Suspicion of aspiration	
Do you have to cough while drinking?	1.04
Do you have to cough while eating? Before drink/after swallowing?	1.32
Do you have to choke while eating/drinking?	1.63
Do you have to cough while choking?	3.08
Are you able to cough?	3.17
Do or did you suffer from pulmonary complications?	1.33
How do you drink? Out of a bottle/from a spoon/by a straw?	2.84
Is the symptom connected with respiratory problems?	2.8
Basic pathway III: Globus sensation	
Do you suffer from globus sensation or other related symptoms?	2.21
Are your symptoms present while you eat/without eating/both?	1.83
Do you suffer from a problem in your throat?	2.52
Do you feel a lump in your throat?	1.7
Do you feel an urge to clear your throat?	1.79
Do you suffer from too much phlegm in your throat?	2.46
Basic pathway IV: Non-cardiac chest pain	
Do you feel pain behind the sternum after a swallow?	1.96
Do you suffer from non-cardiac chest pain or related symptoms?	3.8
Do you suffer from heartburning sensations?	1.76
Do you suffer from reflux?	1.75
Effect of live	
Did you loose weight?	1.12
What is your body mass index?	3.67
Do you suffer from any mood changes?	3.13
Did other changes occur, e.g., in speech, walking, writing, cognition, affection?	*
For how long do the symptoms impair your quality of life?	*
How much is your quality of life impaired by your symptoms?	*
Do you go out to eat and drink with other persons?	*
Can you eat by yourself or need someone´s help?	*
How long does it take for you to finish a meal?	*
Others	
What treatment did you have so far? (medications, previous diagnostic studies, functional swallowing therapy)	*
What do you eat for breakfast/lunch/dinner?	*
Do you use compensatory strategies?	*
Do you suffer from nasal regurgitation?	*
Do you have a dry mouth?	*
Do you feel the food going down when you swallow?	*
Assessment through health professional: Is the patient reliable or not?	*

Numbers indicate the average predictive value assigned by the swallowing experts of the second assessment round

* Indicates questions, that were added as missing in the second interview step, but not rated by all interviewed

In addition, the experts added important questions that were missing after the first interview round. They included 7 general and 13 specific questions (six of them attributed to ‘effect on life’ and seven to ‘others’).

The results showed that 9/25 would definitely use a questionnaire based on the present structure in their daily routine; in a range of 1 (definitely would use the guide) to 6 (definitely would not use the guide), the overall average score of all interviewed experts was 2. Regarding the influence of the patients’ answers on the planning of an upcoming investigation protocol, the specialists assigned an average grade of 1.8, ranging from 1 (very high influence on investigation procedure) to 6 (no influence on investigation procedure).

### Side results

Regarding the average time needed to take the medical history in patients with swallowing disorders, three swallowing experts reported a duration of up to five minutes, six reported a duration of 5–10 min, four reported a duration of 10–20 min, three between 20–30 min, and two reported more than 30 min. Each of the 18 interviewees stated that they documented the history of the patient. Nine of 18 reported using at least one standardized questionnaire about swallowing problems in their clinical routine (Dysphagia handicap index, EAT 10, Mini nutritional assessment, SWAL-QOL, Sydney swallow questionnaire, and locally developed questionnaires). All in all, 10 different questionnaires were mentioned. A history that went well was considered successful if the patient could participate and understood the questions (*n* = 8), the communication led to enough information to formulate a hypothesis (*n* = 8), and there was a correlation between the patient´s history and the results can be found (*n* = 3). The most frequently mentioned reason for a less successful patient–physician communication was shortage of time (*n* = 7).

## Discussion

The goal of this study was to obtain a range of answers from a widespread, diverse group of experts from different disciplines about how to take a medical history in patients with swallowing disorders. Previously, various studies have revealed the relevance of the medical history for making a final diagnosis and showed a high agreement between the diagnosis made after taking the history and reading the referral letter and the final diagnosis [8, 9]. In a study from 2005, Graber et al. found that the majority of cognitive errors in internal medicine occurred due to premature judgment from incomplete data [24]. Clinical evaluation of swallowing is a subjective evaluation to identify possible causes of deglutition disorders, evaluate the risk of aspiration, and decide on further diagnostic tests. As early as 1959, when evaluating dysphagia due to lower esophageal rings, Schatzki stated that, by obtaining a careful history, a strong suspicion of the correct diagnosis could be obtained in 80% to 85% of cases [25]. The role of different swallowing disorders in determining the various causes of dysphagia still remains a challenge. Patients’ subjective experiences of dysfunction are impossible to measure objectively. However, these subjective experiences can be narrated, and selected symptoms and clinically easily assessable variables can help to discriminate different causes of dysphagia [26]. Particularly in the work-up of this patient group, skilled history-taking may lead to differences in planning the diagnostic procedure and in guiding further diagnostic testing.

While 21/25 experts rate the influence of patient history as “high” or “very high,” there are a number of challenges even with a well-tailored questionnaire. Patients’ perception of swallowing disorders is not always reliable, also reflected by the fact that the ability of the patient to participate in the taking of medical history is considered relevant for successful communication. In addition, the speed of onset and mode of progression of specific symptoms may not be helpful in predicting certain diseases. For example, patients with dysphagia associated with benign disease often report weight loss, which may be misleading [14]. For this reason, a general questionnaire that covers a wide range of issues may help to detect patients with swallowing problems that would likely go undetected if diagnosis relied solely on self-reporting.

Another issue is the request to involve the patient in the decision-making process and further diagnostic steps. Patients want an effective dialogue with their physician and authentic caring in their clinical relationship [27–29]. This is reflected by the fact that the majority of experts stated that the ability to establish a therapeutic relationship, as well as an understanding of the patient, and the involvement of the patient and his accompanying persons, were the critical factors for a successful patient–physician communication.

Fast-track investigation of dysphagia has a low success rate in diagnosing esophageal disease, and specialized physicians may facilitate an early diagnosis [14]. General guidelines may be considered too generic to be applied to specific situations. Effective communication differs from situation to situation, reinforcing the need to tailor the communication to the specific situation of each consultation. It has been suggested that specific guidelines be developed for the approach to specific diseases [30].

The advantages of questionnaires include the fact that they are easy to use, do not require a lot of effort compared to the information the physician obtains from the patient, and may be an important tool for evaluating problems and guiding further treatment decisions. Questionnaires for characterizing swallowing disorders vary largely among different institutions, and a review of the literature revealed many different screening tools [31]. This was also seen in our interviews, where nine of 18 interviewed experts indicated the use of at least 10 different questionnaires. Articles that cover tools to identify patients with dysphagia began to be published in 1999 [32]. The increasing number of publications may be explained by the growing presence of speech-language pathologists in the healthcare setting and the progressive concern for the early detection of dysphagia to ensure a safe diet and prevent respiratory and nutritional complications. Most questionnaires cover the oral and pharyngeal symptoms, including items to evaluate aspiration and oropharyngeal dysphagia. This was also seen in our study because questions attributable to suspicion of aspiration and dysphagia were rated as very important, whereas questions categorized as ‘non-cardiac chest pain’ and ‘globus sensation’ were rated as less important. This may be due to the fact that several of the swallowing experts interviewed were specialists in oropharyngeal dysphagia. The intent of this study was to present a collection of questions by experts covering all aspects of the upper gastrointestinal tract.

Radiologists will have contact with patients who have swallowing disorders during their training and career, and could benefit from structured guidance for a comprehensive interview. Determining the correct questions in a clear and simple way during the encounter, to ensure that the patient understands was one of the most important factors named when the experts were asked for examples of successful patient–physician communication. Therefore, a collection of questions that help to explore the diverse symptoms of swallowing disorders comprehensively may serve as an aid for radiologists of all training levels. A better understanding of the causes of swallowing problems, which could be achieved by asking the right questions, helps to customize the investigation of the individual patient, a prerequisite for a correct diagnosis. In addition, recognizing the patients’ problems enhances the interpretation of radiological imaging findings, thus leading to a correct diagnosis and suitable treatment for the patient.

The number of gastrointestinal fluoroscopic examinations has declined during the last 20 years due to the increasing availability of endoscopy and cross-sectional imaging [33]. However, in times of the growing importance and awareness of a value-based imaging care, swallowing studies as safe, non-invasive, and cost-effective diagnostic tests will be appreciated. Controversely, in a busy radiological department time restraints may prohibit a detailed history-taking of all patients, also shown in our study, in which seven experts stated enough time as a significant factor for successful patient–physician communication. The role of the radiologist in this setting is varying in different countries and institutions, ranging from sole responsibility in performing the patient communication and investigation to a more supportive role alongside speech therapists. In most specialized institutions, an interdisciplinary approach is used for an adequate management of patients with swallowing disorders, and the radiologist has preliminary information about the patient´s history.

The experts interviewed reported a quite long time period for obtaining information about the individual patient´s symptoms. A detailed history may be important for several reasons: the accuracy of patient´s symptom localization in dysphagia is not precise [34] and a significant number of patients tend to localize symptoms of distal esophageal pathologies to the neck [35]. Patients may also present for the first time in a radiological department without known aspiration and unspecific symptoms. For these reasons, radiological examinations should be performed by or under the supervision of a licensed physician at the site with understanding of the wide spectrum of symptoms of swallowing disorders, of its correct evaluation and estimation of the patient´s clinical symptoms [36] to guarantee safety of the investigation and to tailor the individual examination correctly. Despite time restraints, history-taking should comprise a brief cover of all subgroups of the general and specific questions to enable understanding of the patients´ problems in its entirety, to include important investigation steps and to correlate the symptoms with radiological findings.

One limitation of this study is that the respondents of the first interview round were not completely identical with the experts of the second survey. Nevertheless, all experts of the second study round were dedicated experts in examining patients with swallowing disorders, with comparable clinical experience and working together with specialists in interdisciplinary deglutology centers.

Although the number of experts is not very high, the topic of a comprehensive history-taking is important, and the presented collection of questions may serve as an aid in taking the history in this selected patient group. Another limitation is the fact that asking these questions is only suitable for patients who are not limited by neurological factors and are able to understand the content of the questions. We waived a third interview round, since the main aim of this study was not reaching consensus, but generated information about the experts´ way of obtaining medical history. Further research is required for consensual creation and validation of a multidisciplinary questionnaire for patients with swallowing disorders.

## Conclusion

This survey presents a collection of the methods used by interdisciplinary experts to perform the clinical interview in patients with swallowing disorders and a weighting of varying questions as an approach to this complex clinical challenge. It may contribute to improve the technique of radiologists of all training levels to take the history in patients with swallowing disorders in order to tailor the examination protocol of the individual symptomatic patient. Although radiologists often do not take a full history in an interdisciplinary setting, it is still important to know the relevant questions across the boundaries of individual disciplines to tailor the radiological investigation optimally and correlate the symptoms to the radiological findings.

## Appendix 1: Interview Manual

1. Initiate the interview: Explain procedure, take contact information.
2. Background information for sampling: What is your profession? What is your specialization?
3. Key question: How do you take the history of patients with swallowing disorders? Do you have a standard procedure?

Background information to better understand answers to the key question:

4. How long does it take you to take a patient´s history?
5. Do you document the history of the patient?
6. Do you use questionnaires routinely? If so, which ones?
7. What is the spectrum of your patients? How many patients do you see?
8. When do you have the feeling that the history you took went well?
9. When do you have the feeling that the history you took did not went well?
10. What would you say is the primary purpose of patient–physician communication? How would you summarize the general aim of a patient–physician communication?
11. Please think of a very successful or very good patient–physician communication. What exactly have you or the physician you observed, done or thought to make the situation very successful or very good?
12. Please think of a less successful or even a bad patient–physician communication. What have you, or the person you observed, done to make it a less successful or even a bad communication?
13. What do you think, or what crosses your mind during a patient–physician encounter?
14. What do you pay attention to regarding yourself or your dialogue partner during a patient–physician communication?
